# Accelerated *in vivo* epidermal telomere loss in Werner syndrome

**DOI:** 10.18632/aging.100315

**Published:** 2011-04-25

**Authors:** Naoshi Ishikawa, Ken-Ichi Nakamura, Naotaka Izumiyama-Shimomura, Junko Aida, Akio Ishii, Makoto Goto, Yuichi Ishikawa, Reimi Asaka, Masaaki Matsuura, Atsushi Hatamochi, Mie Kuroiwa, Kaiyo Takubo

**Affiliations:** ^1^ Research Team for Geriatric Pathology, Tokyo Metropolitan Institute of Gerontology, Tokyo 173-0015, Japan; ^2^ Department Medical Technology, Faculty of Medical Engineering, Toin University of Yokohama, Yokohama 225-8503, Japan; ^3^ Division of Pathology, Cancer Institute, The Japanese Foundation for Cancer Research, Tokyo 135-8550, Japan; ^4^ Bioinformatics Group, Genome Center, The Japanese Foundation for Cancer Research, Tokyo 135-8550, Japan; ^5^ Department of Dermatology, Dokkyo University School of Medicine, Tochigi 321-0293, Japan; ^6^ Department of Pathophysiology, Yokohama College of Pharmacy, Yokohama 245-0066, Japan

**Keywords:** telomere, terminal restriction fragment, Werner syndrome, WRN helicase, skin, muscle, premature aging, segmental progeroid syndrome

## Abstract

Many data pertaining to the accelerated telomere loss in cultured cells derived from Werner syndrome (WS), a representative premature aging syndrome, have been accumulated. However, there have been no definitive data on *in vivo* telomere shortening in WS patients. In the present study, we measured terminal restriction fragment (TRF) lengths of 10 skin samples collected from extremities of 8 WS patients aged between 30 and 61 years that had been surgically amputated because of skin ulceration, and estimated the annual telomere loss. Whereas the values of TRF length in younger WS patients (in their thirties) were within the normal range, those in older WS patients were markedly shorter relative to non-WS controls. Regression analyses indicated that the TRF length in WS was significantly shorter than that in controls (*p* < 0.001). Furthermore, we found that TRF lengths in muscle adjacent to the examined epidermis were also significantly shorter than those of controls (*p* = 0.047). These data demonstrate for the first time that *in vivo* telomere loss is accelerated in systemic organs of WS patients, suggesting that abnormal telomere erosion is one of the major causes of early onset of age-related symptoms and a predisposition to sarcoma and carcinoma in WS.

## INTRODUCTION

Werner syndrome (WS) is an autosomal recessive disorder characterized by premature onset and an accelerated rate of development of major geriatric diseases, including atherosclerosis, diabetes mellitus, osteoporosis, cataract and menopause, and predisposition to sarcoma and carcinoma [1]. Atrophy of the skin and premature graying of the hair are also common in WS patients. The characteristics that define WS among several other premature aging syndromes are that affected individuals show normal growth and development until adolescence, after which the various symptoms appear segmentally in an individual-specific manner [2]. The WS gene (*WRN*) has been mapped to the short arm of chromosome 8 [3], and identified as a RecQ-type DNA helicase-encoding gene [4]. About 75% of WS cases have been reported in Japan.

Human somatic cells are known to have a limited proliferative life span (the Hayflick limit) when serially cultured *in vitro* [5, 6]. When they approach this limit, they cease to replicate and exhibit a state of replicative senescence. The human telomere complex consists of 6-12 kbp of a simple repeating sequence of 6 bases, TTAGGG [7], along with associated proteins [8] located at the ends of chromosomes, and is thought to protect them from being recognized as broken or fused [9]. Decrease of telomeric DNA in human fibroblasts as a function of serial passage during *in vitro* aging was first demonstrated by measurement of terminal restriction fragment (TRF) length [10]. Subsequently it was shown that the proliferative capacity of human fibroblasts *in vitro* could be extended by transfection with the enzyme telomerase [11]. These two lines of evidence led to the telomere hypothesis of cellular aging [12], which in turn was extrapolated to individual aging. However, since human dermal fibroblasts [13], epidermis [14] and other tissues [15] of individuals aged 90-100 years still have TRF lengths of 6-7 kilobase pairs (kbp) and retain proliferative capacity for about 20 doublings, they appear not to have reached the limit of their proliferative capacity. Indeed, Cristofalo et al. reported that there was no significant decline in the proliferative potential of human fibroblasts *in vitro* with age [16]. Hence, recurrent arguments have arisen [17, 18], and these have still not been settled.

Because of its particular characteristics described above, WS has been attracting the attention of gerontologists. Initially, before a link between WS and telomere was identified, cultured dermal fibroblasts from WS patients were found to undergo accelerated rates of decline in their replicative potential and to exhibit a variety of chromosomal translocations and deletions [19, 20]. More recently, the relationship between WRN gene deficiency and the telomere has been investigated extensively. TRF length in fibroblasts from individuals in the early phase of WS has been shown to be comparable with that in cells from normal individuals, but that subsequently accelerated TRF shortening occurs [21]. B lymphoblastoid cells from WS patients demonstrate anomalous increases and decreases of their TRF length during long-term culture after being transformed by Epstein-Barr virus, but finally fail to become immortalized [22]. Although many data have been accumulated using *in vitro* proliferation systems, and the notion of accelerated telomere shortening in premature aging syndromes has been widely accepted, few data on the telomere lengths of cultured cells other than dermal fibroblasts and lymphocytes of WS patients have been obtained. Furthermore, details of telomere shortening *in vivo* in WS and its relationship to the clinical symptoms have been lacking.

In this study, we investigated TRF length in the extremities of WS patients at various ages (range: 30 to 61 yr). All of these patients suffered from skin ulceration, which is a common complication of WS [23], and we attempted to ascertain whether age-related TRF shortening in skin (typical proliferating tissue) and muscle (typical post-mitotic tissue) from WS patients occurs at a rate different from that in normal individuals.

## RESULTS

### Histopathological features of tissue samples

We examined 10 epidermal samples collected from extremities of 8 WS patients aged between 30 and 61 years that had been surgically amputated because of skin ulceration. Their ethnicity was Japanese.We also analyzed four samples of muscle tissue from the zone adjacent to the examined epidermis. Details of the data including the genotypes of the patients are shown in Table 1. As controls, 56 skin samples and 14 muscle samples from autopsy cases unrelated to WS were analyzed.

**Table 1.** Characteristics of the Werner syndrome patients and samples analyzed in this study and their TRF length values

Case^a^ no.	Age [years]	Sex^b^	Genotype^c^ of WRN mutation	Body part sample derived	Skin sample analyzed	TRF length in skin^d^ HinfI digested [kbp]	TRF length in skin^d^ RsaI digested [kbp]	Muscle sample analyzed	TRF length in muscle^d^ HinfI digested [kbp]
WS-1	39	M	4/4	lower leg ankle	+	12.3	11.5	+	14.5
WS-2	43	M	4/4	lower leg knee	+	13.0	12.3	+	13.3
WS-3	62	M	6/6	foot toe	+	8.9	8.0		
WS-4(1)	37	M	4/4	lower leg	+	11.5	11.1		
WS-4(2)	43	M	4/4	upper arm	+	12.8	12.1		
WS-5	30	F	n.d.^e^	upper arm	+	13.2	12.2		
WS-6	42	F	4/4	wrist	+	10.5			
WS-7(1)	41	M	1/4	lower leg	+	11.1	10.4	+	10.1
WS-7(2)	44	M	1/4	lower leg	+	9.2		+	8.5
WS-8	61	M	4/4	lower leg ankle	+	7.1	5.9		

a Single samples were obtained from 6 patients and 2 samples from 2 patients (WS-4, WS-7) at different ages.

b F: woman, M: man.

c Genotyping of mutated WRN gene was performed using TaqMan© SNP Genotyping Assays system (Applied Biosystems).

d Mean of median values.

e n.d.: not determined.

**Figure 1.**
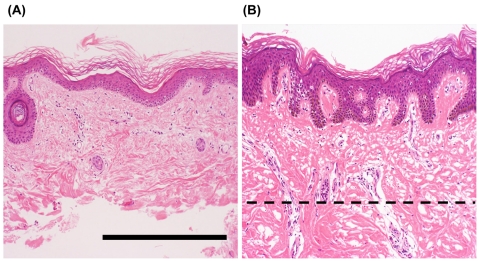
Representative histological features of the skin from patients with Werner syndrome (WS) Representative images of skin specimens from WS patients, stained with hematoxylin and eosin. The left panel (**A**) shows a sample of skin after removal of dermal tissue with a scalpel, and the right panel (**B**) shows a sample before such treatment. Dashed line indicates the treatment border. Figures **A** and **B** show microscopic views of skin from the ankle of a 43-year-old man (WS-2) and the lower leg of a 41-year-old man (WS-7), respectively. Remarkable atrophy of the epidermis and dermis is evident. Mild hyperkeratosis, mild dermal hyalinization, and atrophy of the skin appendages (hair follicles) are present in both cases, with marked flattening of the rete ridges in (**A**). No marked inflammatory cell infiltration is evident in either case. Scale bar, 50 μm.

To define the histopathological characteristics of the skin samples, we performed microscopy analyses. Figures 1A and B show representative microscopic views of the skin tissues from WS patients. The sample on the left (A) is a piece of skin tissue from which dermal tissue had been removed with a scalpel, and the sample on the right (B) is one that was not subjected to such treatment. To determine the representative cell species in each tissue, we counted the numbers of nuclei. The numbers of keratinocytes and non-keratinocytes (including dermal fibroblasts, capillary endothelial cells, and lymphocytes) in section A were 3.3 × 10^2^ (18^2^) and 1.2 × 10^2^ (11^2^), respectively, and those in section B were 5.7 × 10^2^ (24^2^) and 2.2 × 10^2^ (15^2^), respectively. Hence, the ratio of keratinocytes to non-keratinocytes in the samples from which genomic DNA was extracted was 18^3^: 11^3^ in (A) and 24^3^: 15^3^ in (B), thus being approximately 4:1 in both cases. Similar values were obtained in the other cases of WS. Consequently, keratinocytes were considered to be a suitable representative cell type for each skin sample. With regard to muscle tissues, myocytes were the representative cell type.

### Assessment of DNA sample quality by pulse-field gel electrophoresis

**Figure 2.**
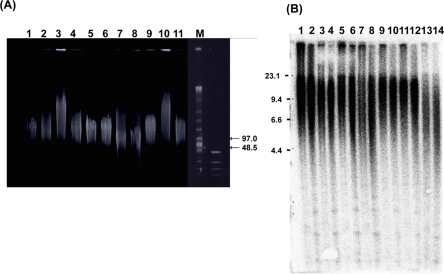
Genofield gel electrophoresis of genomic DNA and Southern blot analysis of samples from WS patients (**A**) Genofield gel electrophoresis of undigested genomic DNA from the skin and muscle was assessed for DNA quality. Samples were applied to a gel as follows; lane 1: WS-5 skin, lane 2: WS-4(2) skin, lane 3: WS-1 skin, lane 4: WS-7(1) skin, lane 5: WS-2 skin, lane 6: WS-7(2) skin, lane 7: WS-8 skin, lane 8: WS-3 skin, lane 9: WS-1 muscle, lane 10: WS-7(1) muscle, lane 11: WS-2 muscle, respectively. The right lanes (M) show size markers. All 11 DNA samples in this figure exceeded 50 kbp in size. (**B**) Representative image of Southern blot analysis of 14 DNA samples from the 7 patients. *Hin*fI digests were applied to lanes 1,3,5,7,9,11,13, and *Rsa*I digests were applied to lanes 2, 4, 6, 8, 10, 12,14. Samples were aligned in order of age; lane 1,2: WS-5, lane 3,4: WS-4(1), lane 5,6: WS-1, lane 7,8: WS-7(1), lane 9,10: WS-4 (2), lane 11,12: WS-2, lane 13,14: WS-3, respectively. The median values of TRF length in this experiment were 12.8, 12.0, 11.4, 11.0, 12.1, 11.3, 10.9, 10.2, 12.9, 12.2, 12.7, 12.1, 8.8 and 7.9 kbp, respectively. The left lane shows a size marker. In the WS-4 and WS-7 patients, skin samples were collected twice at different ages (see Table 1). Corresponding relative copy number profiles calculated by the Telometric program are shown as Supplementary data (Figure S1)

To check the quality of the high-molecular-weight DNA samples, all were subjected to pulse-field gel electrophoresis in an undigested state [24] (Figure 2A). All samples were longer than 50 kbp, and qualified for further TRF analysis.

### Southern blot analysis of TRF length in skin

For quantification of telomere lengths, we digested the DNA from 10 skin samples from 8 WS patients using the restriction enzyme *Hinf*I, and also digested the DNA from 8 skin samples from 7 WS patients using the restriction enzyme *Rsa*I, and performed Southern blot analysis (a representative image is shown in Figure 2B) at least three times for each sample. The means of the median TRF lengths from WS patients are summarized in Table 1. A robust correlation with the paired values was observed (not shown), and generally the value of *Hinf*I digests was greater than that of *Rsa*I digests. The means of the sample values after *Hinf*I and *Rsa*I digestion were 11.0 ± 2.0 and 10.4 ± 2.3 kbp, respectively. The difference between them was significant (p <0.0001). We also digested the DNA from control samples using the restriction enzyme *Hinf*I, and analysed their TRF lengths (summarized in Table S1). With regard to DNA sample quality, the Southern blot data obtained from densitometric analysis using the Telometric software package demonstrated no remarkable signals suggestive of DNA degradation (see Supplementary data, Figure S1).

### Rates of TRF shortening with aging in skin tissues from WS patients and normal controls

**Figure 3.**
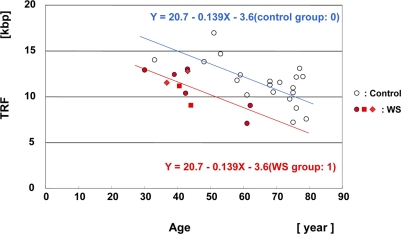
Scatter plot analysis of *Hin*fI-digested TRF length in skin samples from WS patients and controls Multiple regression analysis yielded a regression line for the 8 WS patients (10 samples) (Y = 20.7 − 0.139X (age) − 3.6 (WS group: 1); in red), and a regression line for the non-WS control subjects, aged between 30 and 80 years (n = 21) (Y = 20.7 − 0.139X (age) − 3.6 (control group: 0); control line denoted in blue). The difference of TRF values between the groups was significant. P = 0.00026. The same patient at the ages of 37 and 43 years (WS-4 case: ◆). The same patient at the ages of 41 and 44 years (WS-7 case: ■).

The median values of the *Hinf*I-digested TRF lengths for 10 WS subjects were plotted as a function of age (Figure 3, Supplementary data Figure S2A). The median values of the *Hinf*I-digested TRF lengths for skin in the 56 non-WS control subjects were also plotted as a function of age (Supplementary data, Figure S2A).

To analyze statistically the relationship between the WS and control groups, we applied a multiple regression model that related Y (TRF length) to X_1_ (age) and X_2_ (group), given that the WS group was 1 and the control group 0: Y = β_0_ + β_1_X_1_(age)+ β_2_ X_2_(group). Since the WS patient group did not include neonates and young adults or very elderly individuals, we carried out the analysis using a non-WS control group, omitting neonates, infants, juveniles and the very elderly (n = 21, aged between 30 years and 80 years). This model yielded a regression line indicating that the average annual rate of telomere shortening (β_1_) was 139 bp (*p* <0.0001) and that the difference between the WS and control groups (β_2_) was −3.6 kbp, the group effect being significant (*p* = 0.00026) (Figure 3). Since the difference in the TRF values of 3.6 kbp between the control and WS groups was equal to 0.139 kbp (annual reduction rate)×26 (years), this difference indicated that the TRF lengths in WS patients were equivalent to those in control individuals who were 26 years older.

To assess the TRF attrition rate in each group, we also applied a simple regression model relating Y (TRF length) to X (age): Y = β_0_+ β_1_(X). This yielded a regression line indicating that the average annual rate of *Hinf*I-digested TRF shortening in WS patients was 164 bp (*p* = 0.0047) (Supplementary data, Figure S2A). The *Rsa*I-digested samples were also analyzed in the same way, and this showed that the average annual rate of TRF reduction was 178 bp (*p* = 0.0048) (Supplementary data, Figure S2B). Intriguingly, comparison of the two sets of data from the same individuals at different ages, cases WS-4 and WS-7, revealed that in WS-7 the TRF length decreased as a function of age (−1.9 kbp/3 years), whereas in WS-4 the TRF length increased as a function of age (+1.3 kbp/5 years).

### TRF length in muscle tissues from WS patients and normal controls

**Figure 4.**
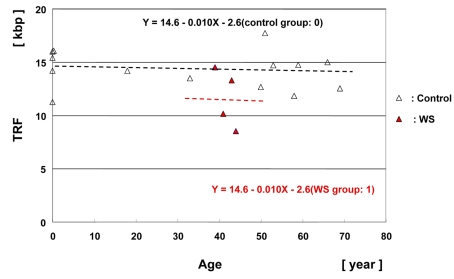
Scatter plot analysis of TRF lengths in muscle samples from WS patients and controls Multiple regression analysis yielded a regression line for the 4 WS patients (Y = 14.6 − 0.010X (age) − 2.6 (WS group: 1); in red) and a regression line for the non-WS control subjects (n = 14), (Y = 14.6 − 0.010X (age) − 2.6 (control group: 0); control line in black).

We next analyzed the TRF lengths of the *Hinf*I digested-DNA samples of muscle tissues from 4 WS patients (shown in Table 1) and control patients (shown in Table S2). The mean of the 4 median values was 11.4 ± 1.0 kbp. The median values of the *Hinf*I-digested TRF lengths in muscle tissue from 4 WS patients and a control (n = 14) were plotted as a function of age (Figure 4). Multiple regression analysis yielded a regression line: Y = 14.6 − 0.010 (X) − 2.6 (group). The TRF lengths in the WS group were significantly shorter than those in the control group by 2.6 kbp (*p* = 0.047).

## DISCUSSION

TRF length measurement by Southern blotting is hampered by the fact that the presence of other cell types always hinders accurate assessment of telomere length in the context of a specific cell type in tissues or organs [25, 26, 27]. In the present study, we separated intact skin tissues from areas showing overt ulceration containing many inflammatory cells, and removed as much connective tissue adjacent to the epidermis as possible. On the basis of histological examination, we confirmed that approximately 80% of each DNA sample was derived from epidermal keratinocytes. Previous studies have employed a variety of methods for detection and calculation of TRF values. The annual telomere reduction rate in normal skin was first reported to be 19.8 bp based on the peak values obtained from densitometric images of TRF [26]. In the present study, we used the software package Telometric and the median TRF values as representatives. The annual TRF reduction rate in the normal controls was 40 bp, which was reasonably compatible with the value of 36 bp obtained in our previous study, in which we used the peak values as representatives [14].

TRF length in every organ of each individual was originally considered to be approximately the same at birth [28], but subsequent measurements of TRF length in cerebral cortex and myocardium showed that it is characteristic to each human individual [11, 29]. The mean TRF length of human systemic organs has been reported to be approximately 13 ± 2 kbp at birth [15, 14, 29]. Since young WS patients are usually quite healthy and have no evident disease [1], it is very difficult to obtain tissues and organs from them. In fact, we were unable to examine TRF lengths in neonatal/infant WS patients, and no data are available in the literature. In view of this situation, the neonatal TRF values for skin in WS patients derived from regression analyses (17.1 kbp based on multiple regression and 18.2 kbp based on simple regression) would seem to be implausible (Figure 3, Figure S2A).

With regard to telomere length dynamics *in vivo*, it has been reported that telomere loss is rapid in the young, but shows slow reduction in the elderly [30, 31, 25]. A longitudinal study of leukocytes from newborn baboons using quantitative fluorescence *in situ* hybridization has provided definitive proof of rapid telomere loss during the very early infant stage [32]. In contrast, our studies have demonstrated that the TRF length in epidermis is well maintained up to middle age (40s -50s), and then gradually decreases. We consider that one of the major causes of this epidermis-specific telomere dynamic is retention of a considerable amount of telomerase activity even in adult epidermal tissue [14]. The profile for WS epidermis also seems to resemble the normal one, except that the time scale is shortened by approximately 25 years (Figure 3). Regarding contradiction of the two sets of data from the same individuals at different ages, cases WS-4 and WS-7, the mechanisms was unrevealed but such anomalous up and down of telomere length seems to resemble the phenomena observed in Epstein-Barr virus transformed B lymphoblastoid cells derived from WS patients [22]. Such anomalous telomere dynamics may universally occur in WS patients segmentally. Contrary to skin, muscle samples demonstrated a flat regression line, suggested that the muscle TRF values for neonatal WS patients are well maintained and typical of a post-mitotic tissue during the period from childhood to adulthood. Hence, in some WS patients, the TRF values could be extremely short even at a very early age.

Cultured fibroblasts from WS patients are characterized by slow growth rates, premature senescence, accelerated telomere shortening and genomic instability [19, 20, 21, 33, 34]. Despite the greater loss of TRF length in cultured cells, it has been reported that the mean TRF lengths of cultured cells from 4 WS patients at earliest passage and senescencewere not shorter than those from 4 normal subjects [21]. Another report has indicated that the TRF length in fibroblasts from a WS patient was shorter than in any of 5 controls subjects examined [35]. Baird *et al.* demonstrated telomere shortening at the chromosome arm level using a PCR-based method in bulk-cultured normal and WS fibroblasts of 99 and 355 bp per PDL, respectively. Data for cloned lines showed that the difference between WS and normal controls was not significant [36]. On the other hand, it has been shown that overexpression of telomerase (hTERT) in WS fibroblast cell lines partially rescues then from accelerated cell senescence [37] and restores genomic stability [38], indicating that one of the consequences of the WS defect is acceleration of normal telomere-driven replicative senescence. It has also been reported that only telomeres replicated by lagging strand synthesis are affected in cells lacking *WRN* [39]. The relationship between this novel topological specificity of the enzyme and accelerated telomere erosion remains to be clarified. In addition, the *WRN* gene product has been shown to have a novel function other than helicase activity, exonuclease activity [40]. Furthermore, not only the *WRN* gene *per se* but also combined functions with various associated molecules, including shelterin (e.g. TRF1, TRF2, POT1) [8, 40] and Flap endonuclease 1 [41, 42], have been shown to be important in telomere metabolism. Recently, it has been reported that Rap1, a member of the shelterin complex, functions in genome-wide transcriptional regulation and NF-κB-dependent signaling [43, 44, 45]. These findings appear to provide a novel perspective of the relationship between extraordinary telomere shortening and the complex symptoms of WS. Our present study has surveyed most expanded WS individuals ever, was partly concordant with previous *in vitro* studies described above, especially the study by Shulz et al. [21] and the bulk-cultured cell data by Baird et al. [36]. Our finding that the TRF lengths in both skin and muscle from WS individuals of the same generation were distributed over a wide range suggest that TRF lengths in WS might be characteristic to each patient, as is the case in normal individuals [15]. The distinctive decrease of TRF lengths in skin and muscle from WS individuals also indicated a tissue-specific effect of *WRN* on telomere maintenance.

To date, the only *in vivo* study of the relationship between a premature ageing syndrome and accelerated telomere attrition was considered to be one demonstrating accelerated TRF shortening in lymphocytes from patients with Down syndrome relative to age-matched controls [46]. Comparison of those findings with our present data revealed some intriguing similarities. Although both sets of data were based on a limited number of samples, several interpretations are possible. If we postulate that there are no differences of TRF length in the early period between Down syndrome and controls, the respective attrition rates and likely explanations could differ. On the other hand, relationships between various progeroid phenomena and defective telomere metabolism have been reported in patients with dyskeratosis congenita, who have mutations of dyskerin or the RNA component of the telomerase gene [47, 48, 49]. Our data suggest an intriguing difference between dyskeratosis congenita and WS, the former affecting mainly proliferating tissues, whereas the latter affects not only proliferating but also post-mitotic tissues. Comparison of these diseases with WS might provide indispensable information.

Since all of the WS patients examined in this study suffered skin ulceration and showed variable TRF lengths, and younger patients in particular possessed TRF lengths compatible with those in the controls, our data did not support telomere shortening in the epidermis as a trigger for the symptoms, and suggested that other pathological cause(s) due to defective *WRN* might play a more significant role in the onset of skin ulceration in WS. Our data demonstrated that such telomere shortening occurred not only in a typical proliferating tissue, epidermis, but also in a post-mitotic tissue derived from mesoderm, muscle. This finding is of particular interest in view of the extraordinarily high incidence of sarcoma at an early age and that of carcinoma at advanced age in WS patients. Understanding the role of single-gene deficiency in the aging process, such as that in WS, could yield valuable information. The present study has provided fundamental data that can serve as a basis for reviewing previously accumulated findings based on *in vitro* cellular analyses from a different perspective.

## MATERIALS AND METHODS

### Subjects

Skin samples from 8 Japanese WS patients were analyzed. Since single samples were obtained from 6 patients and 2 samples from 2 patients (WS-4, WS-7) at different ages, a total of 10 samples were analyzed. All of the skin samples were obtained from extremities that had been amputated due to severe skin ulceration. We also analyzed 4 samples of muscle tissue from the zone adjacent to the examined epidermis. Details of the data including the genotypes of the 8 patients are shown in Table 1. As controls from autopsy cases unrelated to WS, 56 skin samples aged between 0 and 101 and 14 muscle samples aged between 0 and 69 years were analyzed (summarized in Table S1 and Table S2, respectively). The study protocol was approved by the Ethics Committee of Tokyo Metropolitan Institute of Gerontology.

### Preparation and histopathological examination of tissues

The dermis was carefully removed with a scalpel as cleanly as possible from the skin tissues. The tissues samples were examined histopathologically by specialists in anatomical and surgical pathology (K. T., N.I., J.A., Y. I.), and the nuclei were counted.

### Isolation and examination of DNA

Genomic DNA was prepared by the standard method with proteinase K and sodium dodecyl sulfate, followed by repeated manual extraction with phenol-chloroform as gently as possible.

Genomic DNA from all samples was examined their quality by pulse-field gel electrophoresis [24], as described previously [14]. Briefly, they were subjected to the Genofield system (ATTO, Tokyo, Japan), a biased sinusoidal field gel electrophoresis system.

### Measurement of TRF length by Southern blotting

We used the standard method to measure TRF length, as described previously [14]. Briefly, a 5-μg sample of DNA was digested with the restriction enzyme *Hin*fI or *Rsa*I (Roche Diagnostics, Germany). TRF was visualized with a (TTAGGG)_4_ probe labeled with [γ-^32^P]ATP. The TRF image was analyzed using a BAS-2500 Mac image analyzer (Fuji Photo Film) and the Image Reader program (version 1.1, Fuji Photo Film). Representative values of TRF length (median, mean, mode, SIR) were determined using the Telometric software package [50].

### Statistical analyses

Differences in the values were assessed for significance by Student's *t* test. Relationships between TRF length and age were assessed by simple regression analyses, and relationships among TRF length, age and group (WS versus control) were assessed by multiple regression analyses, respectively.

## SUPPLEMENTARY MATERIALS


