# Supplementary material for: Accelerated in vivo epidermal telomere loss in Werner syndrome
Source: Aging (Albany NY). 2011 Apr 25;3(4):417–29. doi: 10.18632/aging.100315 (PMC3117457; doi:10.18632/aging.100315)
Supplement: Supplementary file 1 [file aging-03-417-s001.pdf]

43. Sfeir A, Kabir S, van Overbeek M, Cellis GB, de Lange T. Loss of Rap1 induces telomere recombination in the absence of NHEJ or a DNA damage signal. Science. 2010; 327:1657-1661.
44. Toe H, Ghosh S, Leusch H, Ghosh A, Wong E, Malik N, Orth A, de Jesus P, Perry AS, Oliver JD, Tran NL, Speiser LJ, Wong M, Saez E, Schultz P, Chanda SK, Verma IM, Tergaonkar V. Telomere-independent Rap1 is an IKK adaptor and regulates NF-kB-dependent gene expression. Nat Cell Biol. 2010; 12:758-767.
45. Martinez P, Thanasoula M, Carlos AR, Gómez-López G, Tejera AM, Schoeftner S, Dominguez O, Pisano DG, Tarsounas M, Blasco MA. Mammalian Rap1 controls telomere function and gene expression through binding to telomeric and extratelomeric sites. Nat Cell Biol. 2010; 12:768-780.
46. Vaziri H, Schächter F, Uchida I, Wei L, Zhu X, Effros R, Cohen D, Harley CB. Loss of telomeric DNA during aging of normal and trisomy 21 human lymphocytes. Am J Hum Genet. 1993; 52:661-667.
47. Heiss NS, Knight SW, Vulliamy TJ, Klauck SM, Wiemann S, Mason PJ, Poustka A, Dokal I. X-linked dyskeratosis congenita is caused by mutations in a highly conserved gene with putative nucleolar functions. Nat Genet. 1998; 19:32-38.
48. Vulliamy T, Marrone A, Goldman F, Dearlove A, Bessler M, Mason PJ, Dokal I. The RNA component of telomerase is mutated in autosomal dominant dyskeratosis congenita. Nature. 2001; 413:432-435.
49. Vulliamy TJ, Marrone A, Knight S, Walne A, Mason PJ, Dokal I. Mutations in dyskeratosis congenita: Their impact on telomere length and the diversity of clinical presentation. Blood. 2006; 107:2680-2685.
50. Grant JD, Broccoli D, Muquit M, Manion FJ, Tisdall J, Ochs MF. Telometric: A tool providing simplified, reproducible measurements of telomeric DNA from constant field agarose gels. BioTechniques. 2001; 31:1314-1318.

SUPPLEMENTARY FIGURES

（upper：*Hin*f I-digests、lower：*Rsa* I-digests）

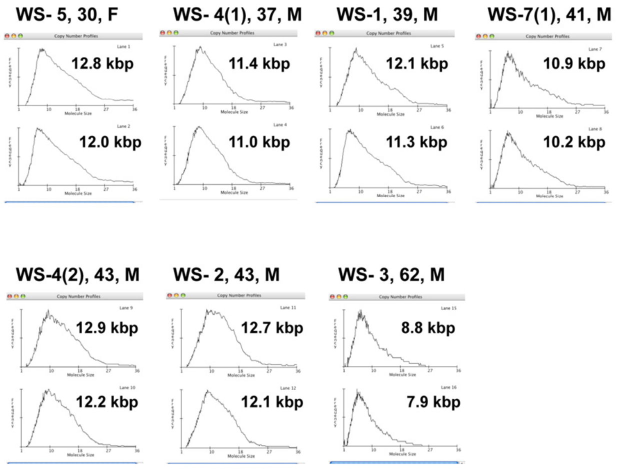

Figure S1. Representative TRF profiles obtained using the Telometric software package. Copy number profiles: plots of the relative copy number versus TRF length corresponding to the data in Figure 2B. Age (shown after case name): years old; F: woman; M: man; kbp: kilobase pairs.

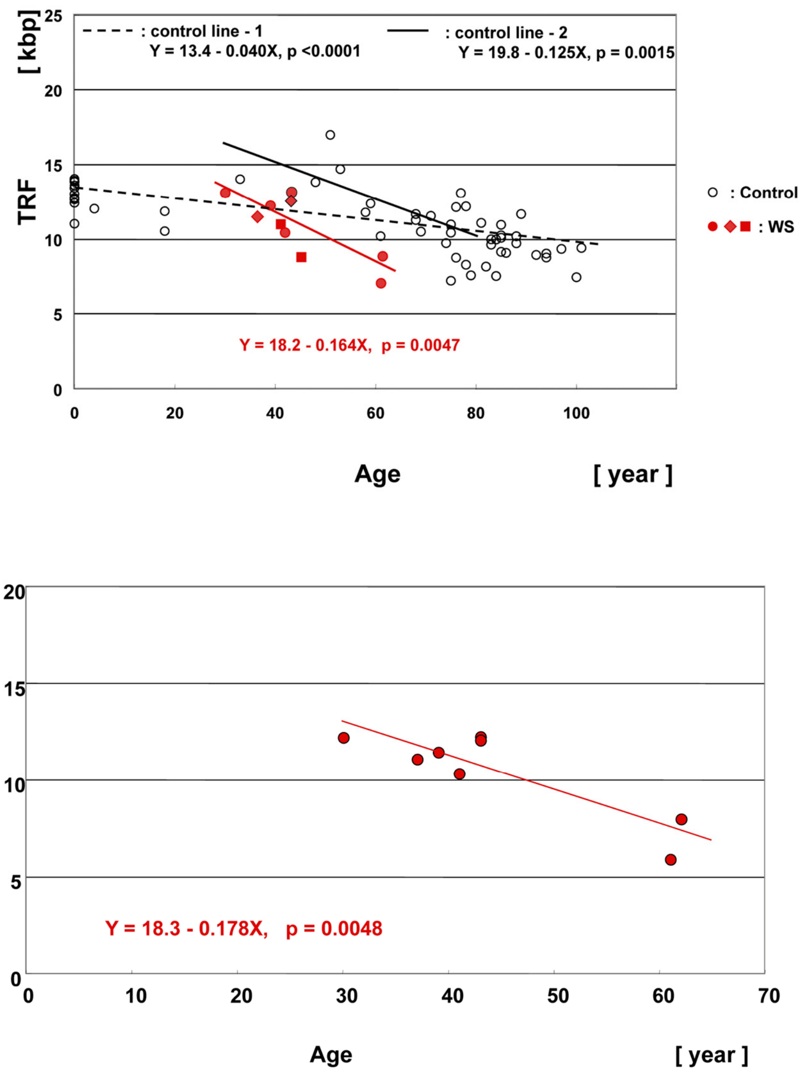

Figure S2 Scatter plot analysis of TRF length in skin samples from WS patients and controls. (A) Simple regression analyses yielded a regression line indicating that the average annual rate of TRF shortening in WS patients was 164 bp (p = 0.0047). The median value of the *Hinf*I-digested TRF lengths for skin in the 56 non-WS control subjects was also plotted as a function of age. The simple regression analysis yielded a regression line (control line-1) indicating that the average annual rate of TRF shortening was 40 bp (p <0.0001). When we performed the analysis again using a non-WS control group, omitting neonates, infants, juveniles and the very elderly (n = 21, aged between 30 years and 80 years), we obtained a regression line (control line-2) indicating that the average annual rate of TRF shortening was 125 bp (p = 0.0015). From these scatter plot analyses, we found that the TRF lengths for younger WS patients (30s and 40s) were situated around control line-1, whereas those for the patients overall were located under control line-2. **(B)** By simple regression analysis, we obtained a regression line for the *Rsa*I-digested TRF length for 7 WS patients (8 samples) (p =0.0048).

SUPPLEMENTARY TABLES

Table S1. Characteristics of the control patients and their skin TRF lengths

Patient ID	Age [years]	Sex[a]	TRF length[b] of skin[c] [kbp]	Cause of death[d]
1	0	M	13.9	
2	0	F	13.1	
3	0	M	11.1	13 trisomy
4	0	M	12.7	
5	0	F	12.5	
6	0	F	12.8	
7	0	F	12.7	
8	0	F	14.0	
9	0	F	13.5	
10	0	F	14.1	
11	0	M	13.9	
12	0	M	13.6	
13	4	F	12.1	
14	18	M	10.6	
15	18	M	11.9	Malignant melanoma of meninx
16	33	M	14.0	Colon cancer, Pancreatic cancer
17	48	M	13.8	
18	51	F	17.0	Cerebral infarction
19	53	F	14.7	Ovarian cancer
20	58	M	11.8	Lung cancer
21	59	M	12.4	Gastric cancer
22	61	M	10.2	
23	68	F	11.7	
24	68	M	11.3	Myocardial infarction
25	69	M	10.5	
26	71	M	11.6	Alcoholic liver cirrhosis
27	74	M	9.8	Sepsis
28	75	M	7.2	Gastric cancer
29	75	F	11.0	Liver cirrhosis
30	75	F	10.5	Myelodysplastic syndrome
31	76	F	12.2	Pneumonia
32	76	F	8.8	
33	77	F	13.1	Aspiration pneumonia
34	78	M	12.2	Acute myelogenous leukemia
35	78	F	8.3	
36	79	M	7.6	Acute myeloblastic leukemia
37	81	F	11.1	Malignant lymphoma
38	82	F	8.2	Acute myelogenous leukemia
39	83	F	10.0	Adult T cell leukemia
40	83	F	9.7	Motor neuron disease
41	84	M	10.0	Gastric cancer
42	84	F	7.6	Myocardial infarction
43	85	F	9.2	Brain hemorrhage
44	85	F	11.0	Gastric cancer
45	85	F	10.3	Cancer of gall bladder
46	85	M	10.1	Cancer of papilla Vateri
47	86	F	9.1	Colon cancer
48	88	F	10.2	Rupture of aneurysm
49	88	F	9.8	Cerebrovascular diseases
50	89	F	11.7	Rupture of aneurysm

51	92	F	9.0	Malignant lymphoma
52	94	F	8.8	Pneumonia
53	94	F	9.1	Aortic stenosis
54	97	F	9.4	Aspiration pneumonia
55	100	M	7.5	Chronic obstructive lung disease
56	101	F	9.4	Gastric cancer

a. F: woman, M: man.
b. Median value of TRF was used as representative.
c. Samples of skin were obtained from abdominal wall near navel.
d. Diagnosis at autopsy.

Table S2 Characteristics of the control patients and their muscle TRF lengths

Patient ID	Age [years]	Sex[a]	Median value [b] of muscle[c] TRF length [kbp]	Cause of death[d]
1	0	F	15.4	
2	0	M	11.2	13trisomy
3	0	M	16.0	
4	0	M	14.2	
5	0.3	F	16.1	
6	18	M	14.2	Malignant melanoma of meninx
7	33	M	13.5	Colon cancer, Pancreatic cancer
8	50	M	12.7	Interstitial pneumonia
9	51	F	17.7	Cerebral infarction
10	53	F	14.7	Ovarian cancer
11	58	M	11.8	Lung cancer
12	59	M	14.7	Gastric cancer
13	66	M	15.0	Brain hemorrhage
14	69	M	12.5	Subarachnoideal hemorrhage

a. F: woman, M: man.
b. Median value of TRF was used as representative.
c. Samples of muscle tissue were obtained from skeletal muscle of thigh.
d. Diagnosis at autopsy.
